# Engineered Lateral Roughness Element Implementation and Working Fluid Alteration to Intensify Hydrodynamic Cavitating Flows on a Chip for Energy Harvesting

**DOI:** 10.3390/mi11010049

**Published:** 2019-12-30

**Authors:** Moein Talebian Gevari, Ali Hosseinpour Shafaghi, Luis Guillermo Villanueva, Morteza Ghorbani, Ali Koşar

**Affiliations:** 1 Sabanci University Nanotechnology Research and Application Center, Tuzla, 34956 Istanbul, Turkey; moeint@sabanciuniv.edu (M.T.G.); alih@sabanciuniv.edu (A.H.S.); morteza@sabanciuniv.edu (M.G.); 2 Faculty of Engineering and Natural Science, Sabanci University, Tuzla, 34956 Istanbul, Turkey; 3 Department of Mechanical Engineering, École Polytechnique Fedérale de Lausanne, CH-1015 Lausanne, Switzerland; Guillermo.Villanueva@epfl.ch; 4 Department of Biomedical Engineering and Health Systems, KTH Royal Institute of Technology, SE-141 57 Stockholm, Sweden; 5 Center of Excellence for Functional Surfaces and Interfaces for Nano-Diagnostics (EFSUN), Sabanci University, Orhanli, Tuzla, 34956 Istanbul, Turkey

**Keywords:** cavitation on chip, energy harvesting, optimization, parametric effect study, hydrodynamic cavitation, microfluidics

## Abstract

Hydrodynamic cavitation is considered an effective tool to be used in different applications, such as surface cleaning, ones in the food industry, energy harvesting, water treatment, biomedical applications, and heat transfer enhancement. Thus, both characterization and intensification of cavitation phenomenon are of great importance. This study involves design and optimization of cavitation on chip devices by utilizing wall roughness elements and working fluid alteration. Seven different microfluidic devices were fabricated and tested. In order to harvest more energy from cavitating flows, different roughness elements were used to decrease the inlet pressure (input to the system), at which cavitation inception occurs. The implemented wall roughness elements were engineered structures in the shape of equilateral triangles embedded in the design of the microfluidic devices. The cavitation phenomena were also studied using ethanol as the working fluid, so that the fluid behavior differences in the tested cavitation on chip devices were explained and compared. The employment of the wall roughness elements was an effective approach to optimize the performances of the devices. The experimental results exhibited entirely different flow patterns for ethanol compared to water, which suggests the dominant effect of the surface tension on hydrodynamic cavitation in microfluidic channels.

## 1. Introduction

Cavitation has received more and more attention during the last decade due to the potential for its implementation in many applications, such as biomedical ones [1], energy harvesting [2], surface cleaning, and water treatment [3,4,5]. Cavitation is associated with a process involving the nucleation, growth, and implosion of bubbles filled with vapor or gas. Such cavities are generated in a fluid when the static pressure drops below the vapor saturation pressure of the fluid. These bubbles implode violently when encountering a higher pressure region, resulting in high temperature spots. The static pressure drop can be obtained using acoustic waves generated by ultrasonic transducers (acoustic cavitation) or by suddenly reducing the hydrodynamic pressure using geometrical changes, typically by increasing the velocity of the fluid or by boundary layer separation (hydrodynamic cavitation) [6,7]. Hydrodynamic cavitation is known to be more energy efficient because of rather simple configuration and construction of related systems, low energy requirements, and easier scaling up possibility for industrial applications [8,9,10]. 

Hydrodynamic cavitation can be observed when the fluid passes through a physical constriction, such as an orifice plate [11] or a venturi [12]. Orifices are one of the most commonly used geometries as hydrodynamic cavitation generators. Despite the extensive use of orifices, the functions and interactions among different designs, and operating parameters of the resulting cavitation behavior, have not yet been sufficiently understood. The geometry of devices, thermophysical properties of the working fluids, surface configurations, operating temperatures and pressures, and contaminant gases in the fluid play important roles in the cavitation phenomenon. 

The geometry of the flow restrictive element is one of the most important parameters in the hydrodynamic cavitation phenomenon. There are numerous studies on geometrical parameters of cavitation generators (flow restrictive elements), such as venturis and orifice plates, in the literature. For instance, He et al. studied cavitating flows in a diesel injector nozzle and showed that hydrodynamic cavitation was beneficial in the separation process of sprays. It was concluded that the radius of curvature (ROC) of the entrance hole was the most crucial parameter for a nozzle in affecting internal cavitating flow characteristics [13]. Rudolf et al. investigated hydrodynamic cavitation in single and multi-hole orifices. Their experimental results illustrated that using multi-hole orifices was more effective than single-hole orifices due to their low energy dissipation. Moreover, based on their study, multi-hole orifices lead to a significant drop of loss coefficient in cavitating and non-cavitating regimes [14]. Dong et al. experimentally studied the effects of number, arrangement, and ratio of the holes in an orifice plate. They proved that cavitation number was minimal for a diagonal structure, while it was maximal for a radial structure. Besides, the turbulent kinetic energy had the maximum value near the edges, and minimum values were seen at the axial lines [15]. Ranade et al. numerically studied the influence of critical geometrical parameters such as orifice thickness, hole inlet sharpness, and wall angle on cavitation flow patterns. According to this study, orifice geometry had a significant influence on cavitation inception. In contrast with the rounded edged orifice, the sharp edged orifice generated more intense cavitating flows. In addition, the required pressure for cavitation inception was increased by 30%–40% with angled orifice walls in comparison to a straight throat section, and as a result, angled orifice walls were undesirable for intensified cavitation [16]. 

Cavitating flows in micro scale differ from those in macro scale. For instance, experimental investigation of cavitating flows in micro-orifices with a rectangular cross-section showed that cavitating flow patterns in micro-orifices are different from macroscale orifices. It was demonstrated that incipient and choking cavitation numbers, and cavitating flow patterns of micro-orifices are heavily influenced by the size scale [17]. In this regard, Jin et al. numerically studied the effect of the ratio between the length and diameter of micro-orifices. They reported that the vapor cavity increased with an increase in length to diameter *(l/d*) ratio, and there existed an optimum value for *l/d* of a micro-orifice based on both cavitation intensity and flow rate [18]. When sufficiently many cavitation bubbles collapse, they release a high amount of energy downstream of the flow channel. This released energy could be utilized to provide the required power for some applications, such as water treatment, surface cleaning, biomedical applications, and energy harvesting. Energy harvesting with hydrodynamic cavitation flows could, thus, be realized in a cost-effective and environmentally-friendly fashion. Surface topology and geometry of the device are among the most significant parameters, which lead to an increase in the intensity of cavitation flows while causing earlier inception. Moreover, the lower inlet pressure for the inception and intensified cavitating flows will result in a higher amount of energy from the collapse of bubbles and improved performance. In this regard, Ghorbani et al. focused on the effects of surface roughness of microfluidic devices on hydrodynamic cavitation. Their results showed that the roughness on the channel surface led to intensified cavitating flows in comparison with a smooth channel over the same range of flow rate. They also suggested that longer microchannels were suitable for energy harvesting applications because of the penetration of the emerging twin cavities [19]. It should be noted that the entire downstream region of microchannel could be filled with vapor and large vapor bubbles by causing twin cavities, which could be exploited in energy harvesting.

Hydrodynamic cavitation is directly affected by thermophysical properties of the working fluid. Therefore, it is crucial to consider this key parameter in microfluidic devices. There are a few studies addressing the application of other working fluids and its effect on flow patterns. For example, Mossaz et al. [20] reported that the mixture of isopropanol and water could change the cavitation inception from turbulent regime to laminar regime. Low percentage of isopropanol in water is likely to increase the dynamic viscosity so that cavitation inception corresponding to laminar flow could be more easily achieved. Hydrodynamic cavitation of water, ethanol, and refrigerant liquids in micro venturis was investigated by Mishra et al. [21]. They compared the results of R-123 to those of water and observed that lower surface tension of this fluid enhanced bubble formation. In a subsequent study, different types of liquids were utilized to obtain flow patterns at low upstream pressures for similar devices [22]. A rapid transition from inception to choked flow condition, beyond which the flow rate no longer increased with upstream pressure, was observed. Ghorbani et al. [23] used poly(vinyl alcohol) (PVA) microbubbles in water inside microchannels with different geometries and compared the results with those of water. They could show that the devices working with PVA had lower cavitation number in comparison with the case of pure water under the same working conditions. The experimental results from this study also demonstrated that the impact pressure upon bubble collapse was more for the case of PVA. Li et al. [24] investigated the influence of thermodynamic effects in the evaporation and condensation coefficient of the cavitation model using an accurate model based on the Rayleigh–Plesset equation. They developed a computational fluid dynamics (CFD) model for simulation of two airfoil cavitation. Their results indicated that the fluid temperature had an effect on the cavitation intensity around the hydrofoil, and the cavitation became more intense with the increase in the temperature. In order to study the cavitation phenomenon and energy generation from hydrodynamic cavitation, Gevari et al. [25] fabricated micro orifices on chips and tested them with different working fluids, including water and a perfluoropentane (PFC5) droplet-water suspension. Based on their results, cavitation inception occurred earlier for the droplet-water suspension. In the case of droplet-water suspension, supercavitation condition appeared sooner, and as a result, the input energy of the system was minimized, which suggested higher energy efficiency.

Energy harvesting with hydrodynamic cavitation has attracted considerable attention during recent years. However, only few attempts have been made regarding the design and optimization of cavitation on chip devices for the purpose of energy harvesting. In this study, the effects of two key parameters in the design and optimization, namely, working fluid and roughness elements inside the microfluidic device, were investigated. In microscale systems, the surface tension force becomes more dominant over body forces such as gravity because of increasing surface to volume ratio. Consequently, surface roughness significantly affects the flow behavior in microfluidic devices. Furthermore, surface roughness is a vital parameter for multiphase flows, where the inception of the secondary phase is from the surface (i.e., boiling, cavitation, condensation). The surface roughness and cavity size are critical parameters in bubble nucleation. In phase change processes, bubble nucleation and bubble departure frequency increase with surface roughness up to a critical size, beyond which surface roughness has no considerable effect on the nucleation process. In this study, the microfluidic devices with a single orifice were fabricated by conventional microfabrication methods. The microfluidic devices were tested at different inlet pressures, and the cavitating flow patterns were visualized. The performances of the microfluidic devices were evaluated, and wall roughness elements were added to the originally fabricated plain surface microfluidic device. The effect of these elements on cavitation flow patterns was studied, and the optimum design area was obtained. Changing the working fluid to intensify cavitating flows in the extension area was the last step in optimization. Finally, an optimum design was suggested for microfluidic devices for achieving facile and intensified cavitating flows.

## 2. Materials and Methods

The energy release from the collapse of bubbles in cavitating flows could be utilized in many applications, such as water treatment, ones in the food industry, and biomedical ones. Both facile generation and increasing the intensity of cavitating flows to generate more cavitation bubbles are in favor of such applications. In addition, lower inlet pressure for the inception and facile development of cavitating flows could increase the efficiency. Adding roughness elements inside the microfluidic device and changing the properties of the working fluid are two main approaches for the optimization in this article. 

### 2.1. Microfluidic Device Design Parameters and Fabrication

**Figure 1:**
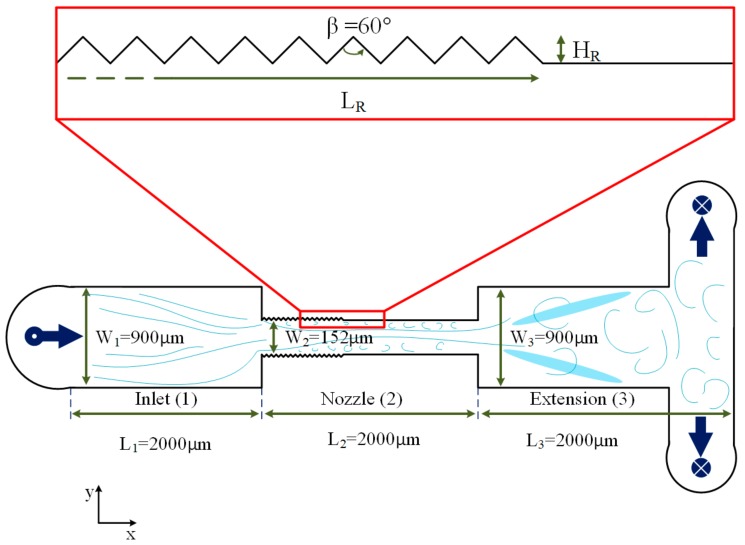
The configuration of the microfluidic device consisting of three main sections—inlet, nozzle, and extension, with wall roughness elements (the total length of roughness elements (*L_R_*) and height of the roughness elements (*H_R_*)).

A typical microfluidic device houses a single orifice high pressure hydrodynamic cavitation system etched on a silicon wafer. The general configuration of the fabricated microfluidic device can be seen in Figure 1. 

The microfluidic device is divided into three main sections; namely, inlet, nozzle, and extension. The working fluid enters the inlet perpendicularly and follows the geometry of the microfluidic device after a 90° rotation. The relatively small cross sectional area of the microchannel section provides the sudden pressure drop of the working fluid, which triggers cavitation. Engineered lateral wall roughness elements are embedded on the walls of the nozzle with different total lengths and heights in equilateral triangular shapes. An extension chamber is located downstream so that the fluid could recover its pressure loss, and the bubble collapse is facilitated. In a previous article, the bubbles collapsed on a thermoelectric generator wall, which was located at the end of the extension to harvest heat from the collapsing bubbles and to generate electricity [25]. As a result, to achieve effective energy harvesting, more bubbles in the extension are desired. Finally, the fluid exits the microfluidic device from two outlets after a 90° rotation. Seven configurations were fabricated with different geometrical parameters of roughness elements. The width of the inlet and extension is kept the same (900 µm), while the width of the microchannel is 152 µm for all the cases. The length of all three sections is 2000 µm. As a result, the total length of the microfluidic device is 6000 µm for all the cases. Three short and narrow microchannels are etched on the silicon substrate along with the fabrication of the main microfluidic device housing pressure ports, where the pressure gauges are integrated to the experimental setup: one at the inlet, one at the nozzle, and one at the extension. The static pressure of the fluid was measured, and the corresponding data points were acquired.

The first microfluidic device has a smooth wall with no roughness. In the other six devices, triangular wall roughness elements are achieved in the microchannel section with different total lengths and heights. The total length of the roughness elements corresponds to one third, half, and total length of the microchannel (2000 µm). The heights of the elements, on the other hand, are 0.1 and 0.01 of the microchannel widths. Table 1 shows the geometrical parameters of microfluidic devices 1 through 7. 

**Figure 2:**
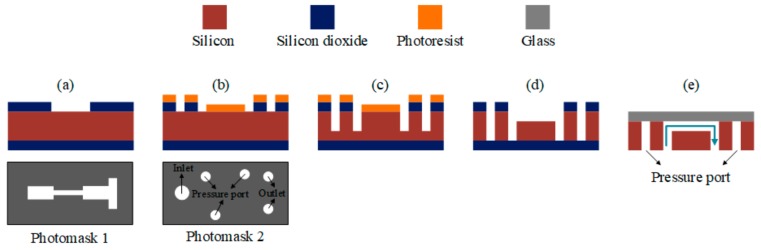
Fabrication process flow. (**a**) The etched silicon dioxide layer after the first photolithography step; (**b**) the etched silicon dioxide layer after the second photolithography step before removing the photoresist; (**c**) the first deep reactive ion etching of the wafer; (**d**) the second deep reactive ion etching of the microfluidic channel after removing the photoresist layer; (**e**) microfluidic device anodically bonded to the glass (the final product).

Standard microfabrication methods were used to fabricate the devices. For this purpose, a 500 nm thick layer of SiO_2_ was grown on a <100> silicon wafer using plasma enhanced chemical vapor deposition (PECVD). A photomask was designed for a photolithography step in the Layout-editor software to make one opening on the substrate corresponding to the whole geometry of the microfluidic device. Reactive ion etching (RIE) of SiO_2_ layer and photoresist removal correspond to Figure 2a. The second lithography step with the second photomask followed by a dry etching step similar to the previous step resulted in Figure 2b. A deep reactive ion etching (D-RIE) process for 200 µm was utilized (Figure 2c). After the photoresist removal, a further 50 µm deep D-RIE led to Figure 2d. It is worth mentioning that a 2 µm thick protective layer of Ti and Al was deposited on the backside of the substrate before the last D-RIE so that it survived the stress applied to the sample due to deep etching. The remaining SiO_2_ layer with the protective Al layer on the back was removed. Finally, the substrate was anodically bonded to Borofloat-33 glass after the Piranha cleaning process (Figure 2e). The resulting microfluidic device was a 50 µm deep single orifice with one inlet and two outlets along with three pressure ports etched on a silicon wafer with glass lead. The total of 5 holes in the microfluidic device were 1 mm in diameter, for which a suitable experimental setup was designed.

### 2.2. The Effect of Lateral Roughness Element Implementation 

The sudden pressure drop in the microfluidic device initiates homogeneous and heterogeneous nucleation in the system. Homogeneous nucleation happens in the bulk fluid, while heterogeneous nucleation happens on the interface of the solid and liquid, no matter how small the solid body is. It can even happen on an external particle floating in the body of the fluid. 

Surface tension (*S*) or surface energy represents the intermolecular forces preventing void generation in the bulk of fluid. To elaborate briefly on the homogeneous nucleation in the microfluidic devices, surface tension is scaled down to the microscopic value of bubbles a few micrometers in size [26,27]. The difference between the interior pressure of a bubble filled with pure vapor ($P_{vap}$) and the surrounding pressure (P), denoted as ($\Delta P_{C}$*),* would appear as Equation (1), which is an indicator for the tensile strength of the liquid:

(1) $$\Delta P_{C}=P_{vap}-P=\frac{2S}{R_{C}}$$

When the surrounding pressure *P* in Equation (1) drops to a level less than ($P_{vap}-2S/R$), the bubbles start growing until a critical radius ($R_{C}$), and consequently, rupture happens. As a result, an amount of energy is needed to overcome the surface tension between the liquid molecules to generate the void. On the other hand, some work is needed to be performed on the liquid (as a control volume) to push the molecules out of the void area. The first term on the right hand side of Equation (2) shows the surface tension energy, while the second term stands for the required work on the control volume:

(2) $$W_{CR}=4\pi R_{C}^{2}S-\frac{4}{3}\pi R_{C}^{3}\Delta P_{C}$$

This equation includes the total energy ($W_{CR})$, which is needed for the bulk fluid to nucleate homogeneous bubbles. Homogeneous bubbles might be confused with heterogeneous bubbles because there exist external, submicron-sized contaminant particles in the liquid facilitating the heterogeneous nucleation. In addition, the contaminant gases in the liquid start to generate bubbles once the pressure drops under a critical value, which might also be confused with homogeneous nucleation of bubbles. As a result, homogeneous bubble nucleation study is rather complicated. 

**Figure 3:**
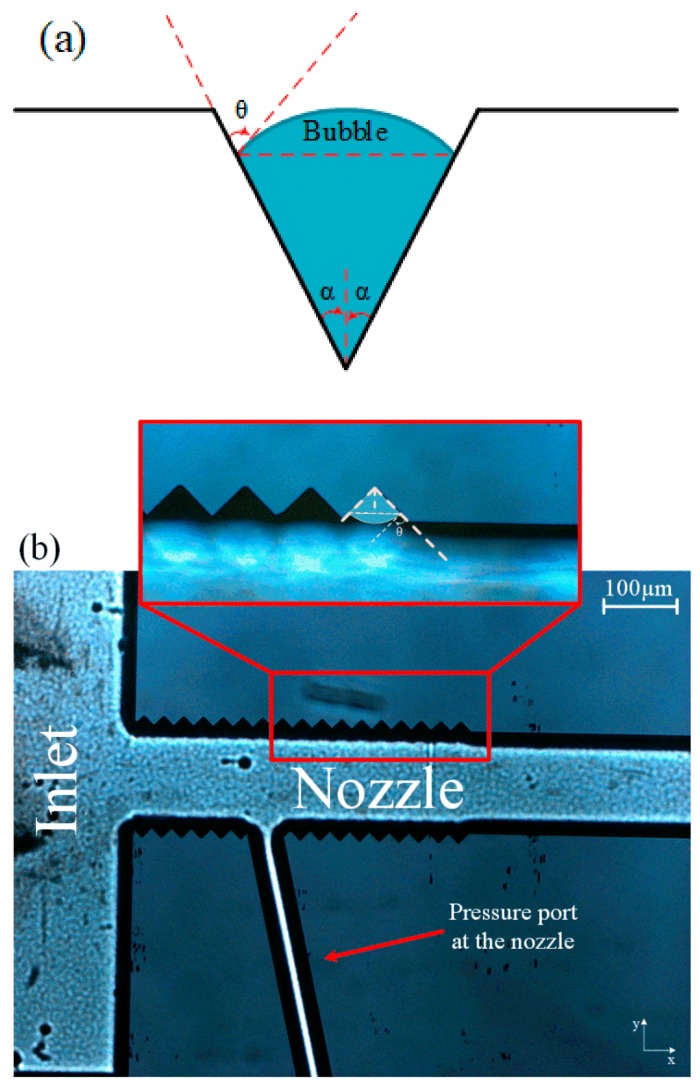
(**a**) Wall roughness elements and bubble (**b**) optical microscopy image of the wall roughness element inside the microchannel.

In the heterogeneous nucleation, bubbles form on the interface of solid and liquid. The tensile strength in heterogeneous nucleation of bubbles is expressed as:

(3) $$\Delta P_{C}=\frac{2Ssin\theta }{R}$$

where *R* is the radius of the generated bubble, and *θ* is the contact angle at the interface of gas, liquid, and solid (Figure 3a). The smaller *θ* reduces the tensile strength of the liquid, which leads to earlier nucleation of the bubbles. The fabricated microfluidic device, which did not exhibit any good performances in the first phase of the optimization (no cavitation), was equipped with wall roughness so that it could reduce the inlet pressure, at which heterogeneous nucleation happens. Figure 3a shows the wall roughness elements on one of the fabricated microfluidic devices. There were two design parameters for adding roughness elements to the microfluidic device which needed to be optimized: the height of the elements (*H_R_*) and total length of the elements (*L_R_*). Six different microfluidic devices were fabricated for this task, as shown in Figure 3b and Table 1.

### 2.3. Experimental Setup Design and Assembly

**Figure 4:**
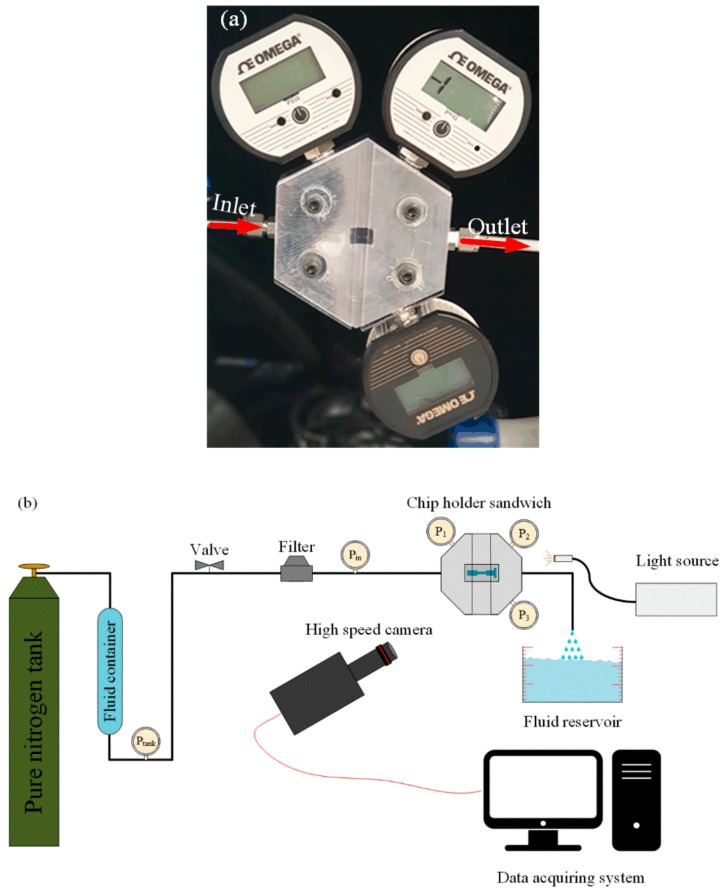
(**a**) Chip holder sandwich (package) with a microfluidic device consisting of three pressure sensors measuring pressure at inlet, nozzle, and extension. (**b**) Schematic of the experimental setup consisting of a high pressure pure nitrogen gas tank pressurizing the working fluid in the container, the chip holder sandwich (package), a proper light source, high speed camera, valves, data recorder, and the fluid reservoir.

The microfluidic device needs to be sandwiched so that it can withstand high pressures ensuring no leakage. For this purpose, a package with 5 holes with the similar depth as the thickness of the microfluidic devices was fabricated so that the fluid could enter from one port and exit from the other. The package was designed in such a way that the pressure gauges could be installed to measure the pressure. The devices were tightened on their groves using Plexiglass to facilitate the flow visualization using a double-shutter CMOS camera with a resolution of 1280 × 800 pixels with pixel size of 0.02 mm. A macro camera lens (type K2 DistaMax) with focal length of 50 mm and *f*-number of 1.2 was employed to capture flow patterns inside the microfluidic device during the experiments. Figure 4a shows the fabricated package with a device. The experimental setup, on the other hand, consisted of a large fluid container (Swagelok, Erbusco BS, Italy), which was connected to a high pressure pure nitrogen tank (Linde Gas, Gebze, Kocaeli, Turkey). The nitrogen flow pressurized the fluid in the container and pushed it into the system. A pressure gauge (Omega) measured the pressure of the system after the container. The working fluid entered the sandwich after passing through a micro T-type filter (Swagelok) with nominal pore size of 15 μm to filter unwanted particles larger than 15 μm. A proper portable point halogen light source aided the flow visualization during the experiments. Figure 4b shows the schematic of the experimental setup. 

## 3. Results and Discussion

In the fabricated microfluidic devices, the heat dissipation upon cavitating bubble collapse was meant to be harvested and converted into electricity by a thermoelectric module installed at the end wall of the extension, where bubbles were targeted to. As a result, intense cavitating flows at lower inlet pressures were to increase the efficiency of the energy harvesting system. In other words, inception and intensified cavitating flows in the microfluidic device were desired to be at lower pressures. As a result, lower inception and cavitation development pressures of cavitating flows constitute the optimization goals of this study. The optimization process was performed in three phases, which are explained in the next sections. 

### 3.1. Phase One: Initial Design and Analysis

The inlet and extension width of the initial design, as mentioned before, was 900 µm, and the width of the microchannel was 152 µm. The widths and lengths of the microfluidic devices were kept constant during the optimization process. Cavitation number, Equation (4), as an indicator to characterize the cavitation phenomena, is considered here as an optimization parameter along with the inlet pressure, at which cavitating flow incepts:

(4) $$\sigma =\frac{\left(P-P_{vap}\right)}{0.5\rho V^{2}}$$

where *P* is the inlet pressure, *P_vap_* is the saturation vapor pressure of the working fluid, ρ is the fluid density, and *V* is the characteristic velocity of the fluid in the microfluidic device, which is calculated at the beginning of the nozzle based on the volumetric flow rate of the system (flow rate/cross sectional area).

Flow pattern images obtained from the high speed camera during the experiments are also compared at different inlet pressures to indicate the cavitation intensity. Achieving more intensity of the generated cavitating flows is of interest. In addition, reaching developed cavitating flows at lower inlet pressures raises the efficiency in possible applications. In conclusion, more intense cavitating flows at a lower inlet pressure are in favor of this optimization. 

The initially designed microfluidic device (device 0) was a plain surface micro orifice with the geometrical features shown in Table 1. The device was installed on the chip holder sandwich (package), and the inlet pressure was gradually increased. The flow patterns were recorded simultaneously during the experiment. For the plain wall configuration, no inception of cavitation was detected over the entire inlet pressure range. The microfluidic devices are durable to inlet pressures of about 7584 kPa. This microfluidic device needed to be optimized.

### 3.2. Phase Two: Wall Roughness

Procedure: The inlet pressure is gradually increased and is kept constant every 350 kPa for a few minutes to make sure that the transient state of the flow pattern passes, and steady state is achieved. The inlet pressure is increased until inception of cavitating flow could be captured by the visualization system of the experimental setup. The cavitation numbers at three different inlet pressures are calculated and compared after the experiments. It should be noted that the inlet pressure is considered as the pressure term in cavitation number (Equation (4)), and the saturation vapor pressure of water at 20 °C, 2333 Pa, is the other pressure term in this equation. The velocity of the fluid is also calculated using the volumetric flow rate at the beginning of the nozzle, where the velocity is maximum.

**Figure 5:**
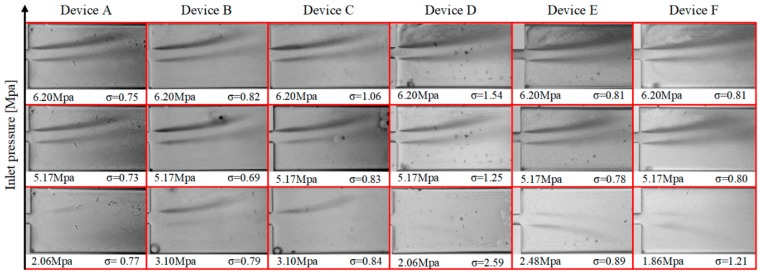
The flow patterns of six microfluidic devices in the extension section of the devices along with the inlet pressure and cavitation numbers.

The density was 998.2 kg/m^3^ in this analysis. Figure 5 shows the cavitation number and the inlet pressure of each microfluidic device and the corresponding flow patterns. The cavitation number in microfluidic devices first decreased and then increased in all the tests. According to Equation (4), the decrease in the value of the cavitation number is as a result of the velocity increase with the inlet pressure. This trend continues until the microfluidic channel flow is choked. Beyond this condition, the increase in the inlet pressure does not change the velocity of the working fluid. Thus, the cavitation number increases, which could be considered as the indicator of the choked flow. The microfluidic devices were tested until this trend was observed for all the cases.

As shown in Figure 5, the extension inception (the onset of cavitating flow in the extension zone of the microfluidic devices) in device A happens at 2.06 MPa, while the same flow pattern is seen at a higher inlet pressure of 3.10 MPa in device C. The difference between these two devices is the total length of the roughness elements. According to this observation, shorter total length of the roughness elements in device A exhibits a better performance compared to device C. In addition, the nozzle inception (the inception of cavitating flow at the beginning of the nozzle) happens at 4.13 MPa, while it never happens in device C. This trend also confirms the better performance of device A compared to device C. On the other hand, the performance of device B resembles the performance of device C in the extension. However, the nozzle inception was recorded at 5.17 MPa in device B, which suggests a relatively better performance compared to device C with no nozzle inception. For the first three microfluidic devices (devices A, B, and C in Table 1), considering the inlet pressure for inception of cavitating flows in the extension section of the microfluidic devices as the optimization goal, it can be concluded that the optimum total length of the wall roughness elements is one third of the total length of the microchannel. Furthermore, the shorter the total length of the lateral roughness elements, the better the performance acquired from the device will be. However, comparing the performances of devices D, E, and F (Table 1) leads to a different conclusion. The difference between this group of the microfluidic devices and the previous three devices is the height of the roughness elements, which is ten times larger. Device F with the longest total length of the roughness elements exhibits the best performance among the tested microfluidic devices. The extension and nozzle inception happens at 1.86 MPa, while the inceptions in the other devices happen at 2.06 MPa and 2.48 MPa, respectively.

**Figure 6:**
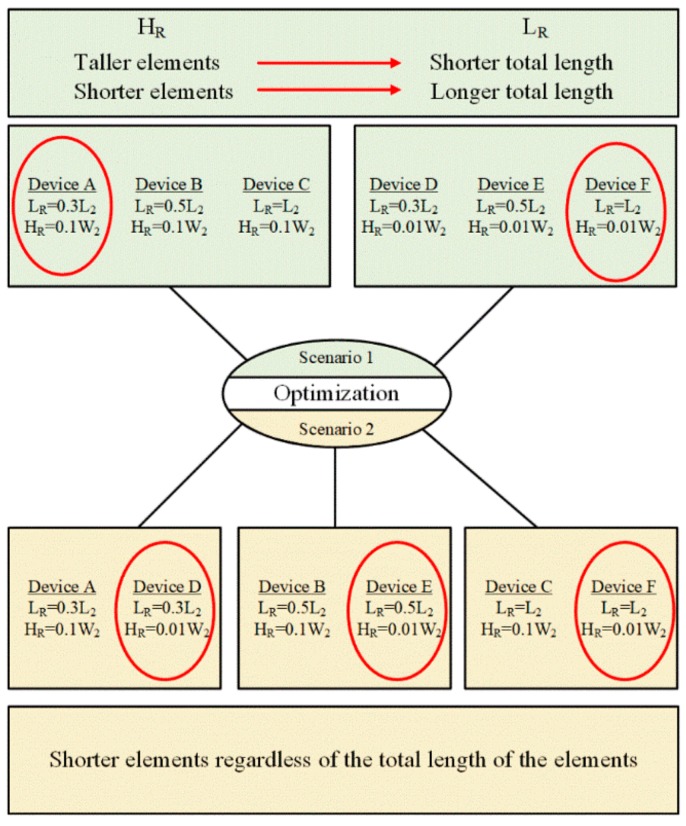
Optimization scenarios and conclusions from the optimum cases in each scenario.

The results of the experimental observations could be interpreted in two scenarios. In the first scenario, the microfluidic devices are divided in two groups. The members of each group have the same roughness element height but different total lengths. In the second scenario, on the other hand, the microfluidic devices are divided in three groups, in which the lengths of the roughness elements are the same but their heights are different. Based on the first scenario, the shorter total lengths of roughness elements for the tall elements, device A, and longer total length for short elements, device F, are in favor of this optimization process (Figure 6). On the other hand, from the second scenario perspective (roughness height), comparing devices A and D, B and E, and C and F, it can be concluded that the shorter elements lead to a better performance, and this conclusion is consistent for all three groups in this scenario. The difference between these pairs of devices lies in the height of the roughness elements. For instance, the roughness height in device C is more than in device F, and the inception happens at 3.10 MPa, while the inception inlet pressure for device F is 1.86 MPa. The other two pairs of the devices follow the same trend in their results. The experimental results for the nozzle inception are also consistent with the extension results. 

After both scenarios, devices C and E could be named as the worst cases for all six microfluidic devices, which will be the interest of the next phase of optimization. The inception of cavitating flows in the extension happens at 3.10 MPa in device C, and the intensity of cavitating flows in the extension is not very high at the highest inlet pressure of 6.20 MPa (Figure 5). In device E, on the other hand, the inception happens at 2.48 MPa, and cavitating flows are not intense in the extension at 6.20 MPa (Figure 5).

**Figure 7:**
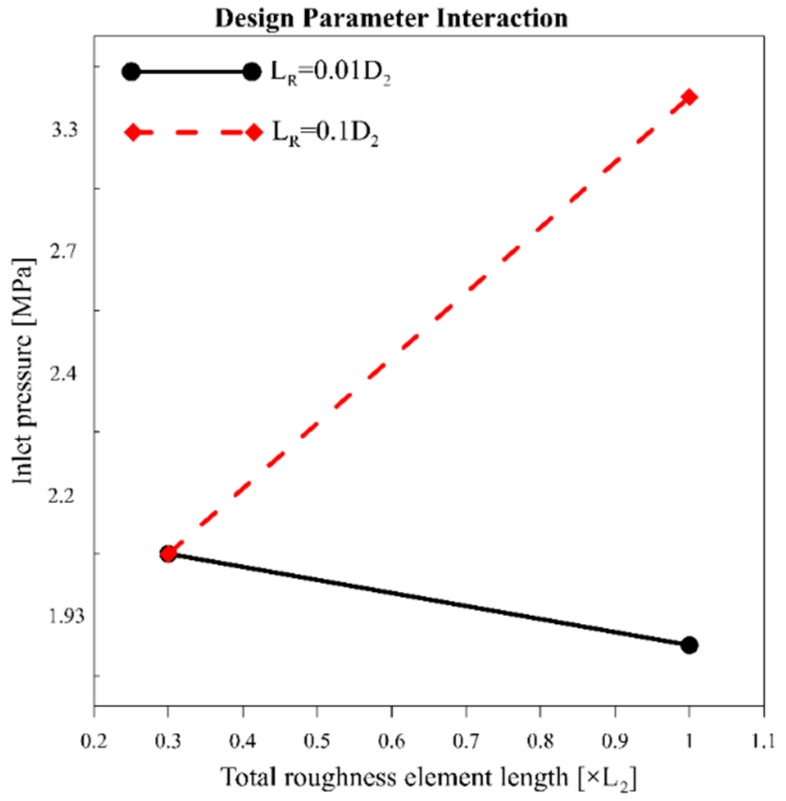
Design parameters interaction plot showing an intense interaction between the wall roughness elements and geometrical dimensions.

The analysis results from the first scenario mark device A as the optimum design, which has a shorter total length (*L_R_*) and taller roughness elements (*H_R_*) on the wall, whereas the second scenario suggests device F as the optimum design, which has the longest total length and the shortest roughness elements. The contradiction between the analysis results shows an interaction between the design parameters. A full factorial design on the microfluidic device considering both length and height of the roughness elements shows this interaction in Figure 7. The steep angle between the parameter lines in the interaction profile bolsters this claim. 

### 3.3. Phase Three: Working Fluid Change to Ethanol

We aimed use the microfluidic device we were working on in an energy harvesting system. As a result, a larger number of bubbles in the extension area was desired so that the thermal energy could be harvested from the collapsing bubbles. The fabricated and tested microfluidic devices in the previous phases did not exhibit good performances in this regard. The extension areas in neither of the cases showed an intense presence of cavitating bubbles, even at high inlet pressures. As mentioned in Section 2.2, the tensile strength of the working fluid plays a critical role in bubble generation. Equation (1) shows the tensile strength of the working fluid, and Equation (2) expresses the required energy to initiate the phase change. Decreasing the surface tension of the working fluid leads to a lower tensile strength and lower energy requirement. The surface tension of water is 72 mN/m at 25 °C, while the surface tension for ethanol is 21.78 mN/m at the same temperature. The lower surface tension of ethanol makes it a good candidate for the optimization of the microfluidic devices; 100% ethanol was used to run new sets of tests on the fabricated microfluidic devices from the previous phase of the optimization. In the second phase of the optimization, the worst performing devices in terms of the cavitation inception were devices C and E. 

**Figure 8:**
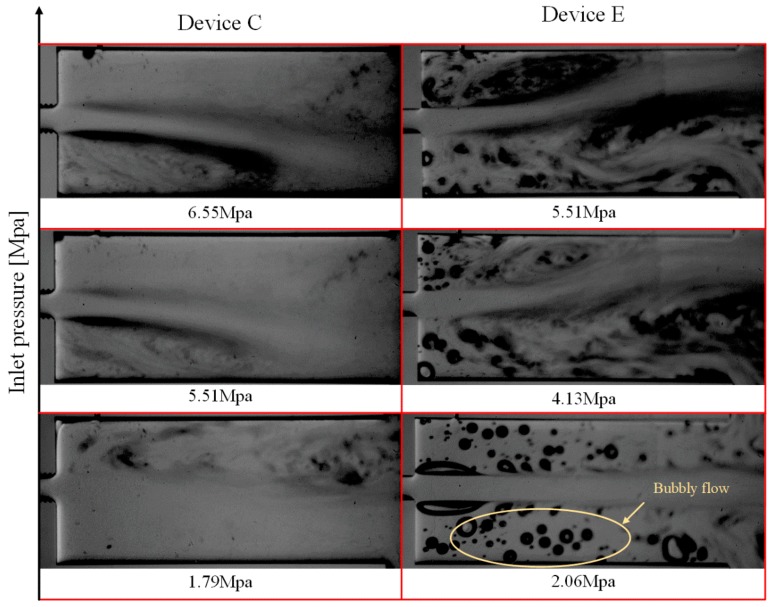
Cavitation flow patterns for devices C and E (worst cases in the previous phase) working with ethanol.

In the case of ethanol as the working fluid, the intensity of cavitating flows in the extension is significantly higher in comparison with the cases with water. The images from the high speed camera show that the number of the bubbles generated in the extension section is significantly higher than that of the case of water at the same inlet pressure. This observation was due to the lower surface tension of ethanol in comparison with water. Apart from the inception pressure difference between ethanol and water, the physics of extension inception are also different. The inception in experiments with water happens gradually with a weak bubble cloud in the extension, while the bubble cloud in the case of ethanol appears suddenly and intensely. In addition, the lower surface tension of ethanol increases its tendency to form bubbly flow rather than a bubble cloud, which could be seen in the experiments with water (Figure 5). Figure 8 shows cavitating flows in the extension of devices C and E working with ethanol.

The inception pressure in device C working with water is 3103 kPa, while this value drops to 1793 kPa for ethanol. The same trend is visible for device E with inception pressure difference of 414 kPa. The reason for this observation could be explained by flow rate expressed as Equation (5) [28]:

(5) $$Q_{cav}=W\;H{\left[\frac{1}{\rho }\frac{P_{out}-P_{vap}}{\frac{W}{wC_{d}}-1}\right]}^{\frac{1}{2}}$$

where the width of inlet and nozzle are denoted as *W* and *w*, respectively; *H* is the depth of the etched channels in the silicon substrate; and *C_d_* is the discharge coefficient of the device.

The flowrate, at which cavitation incepts, is formulated with the geometrical parameters and the thermophysical properties of the working fluid. The vapor pressure of ethanol at 25 °C is 7.83 kPa, and the density is 789 kg/m^3^, while the vapor pressure and the density of water are 3.17 kPa and 998.2 kg/m^3^, respectively. The higher vapor pressure of ethanol in comparison with water decreases the critical flowrate, at which cavitation incepts, which explains the earlier inception of cavitating flows. 

Since the proposed energy harvesting system uses the released heat energy from the collapsing bubbles to generate electricity, ethanol could be nominated as a better working fluid. In this phase of the optimization, the working fluid was changed so that the fluid properties would be in favor of that application. Comparing the flow patterns of ethanol and water, more bubbles in the extension are visible for ethanol. On the other hand, the generated bubbly flow and larger bubbles in the extension release more energy upon collapse. Equation (6) can be employed to find the potential energy of the bubbles generated in the microfluidic device:

(6) $$E_{pot}=\frac{4}{3}\pi R^{3}\left(P_{stat}-P_{vap}\right)$$

where $P_{stat}$ is the static pressure of the surrounding. As can be seen, the potential energy of bubbles ($E_{pot}$) is in direct relation with the third power of the radius of the bubble. More than half of this energy is converted to heat after collapse of the bubbles, which is the target of the proposed system [29]. 

Table 2 summarizes and compares the design and optimization phases in this study. The initially designed microfluidic device did not show a good performance in terms of cavitation generation. In Phase II, wall roughness elements with different heights (*H_R_*) and lengths (*L_R_*) were employed in the nozzle section. The change in the tensile strength of the working fluid facilitates cavitation inception in the extension. The worst cases (performances) in this phase were chosen for the last phase. Ethanol as the working fluid was nominated for Phase III so that the thermophysical differences with water could work in the favor of this optimization goal. The results indicate that the early inception of cavitating flows and its intensification can be achieved by adding lateral roughness elements to the microfluidic devices. Changing the working fluid from water to ethanol also serves for intensification of cavitating flows. Thus, more cavitating bubbles can be generated in the extension, and more energy harvesting from the collapsing bubbles could be attained.

## 4. Conclusions

In this study, single orifice microfluidic devices were designed and fabricated on silicon and were anodically bonded to glass. The fabrication of the devices in silicon ensures their resistance to high pressure flowing flows without having any mechanical failure or geometrical deformation. An experimental setup was designed and assembled in order to visualize cavitating flow patterns and to measure pressures at different points. The initially designed microfluidic device was equipped with six different lateral wall roughness element configurations, and the performances of the configurations were studied experimentally. Cavitation number was used as the major parameter to provide consistent comparisons during the tests. Two scenarios were considered to analyze the results. In the first scenario, the microfluidic devices were divided in two groups with the same roughness element heights but with different total lengths of roughness elements. The results showed that shorter total length leads to an earlier and more intense cavitating flows in the extension for taller roughness elements. In addition, longer total length of the lateral roughness elements increased the performance for shorter roughness elements. In the second scenario, on the other hand, the devices were divided into three groups, while the total lengths of the roughness elements were the same and their heights were different. The experimental results confirmed that shorter roughness elements exhibited a better performance regardless of the total length of the roughness elements. Since the conclusions from two scenarios did not match, a parametric effect study was also performed to examine the interactions among the design parameters. 

In the last phase of optimization, the worst cases from the previous phase were selected for the tests with ethanol as the working fluid instead of water. The results showed more intense cavitating flows at low inlet pressures for these devices due to the thermophysical differences between the working fluids. 

The employment of wall roughness elements inside the microchannel could facilitate cavitation flows. Although the initially designed microfluidic device did not generate cavitating bubbles at any inlet pressure, all the secondarily designed and fabricated devices led to cavitating flows in their extension. Ethanol, due to its thermophysical properties, could considerably enhance the performances of the microfluidic devices. Thus, based on the results from the present study, ethanol could be considered a strong candidate for the working fluid to enhance the intensity of cavitating flows. Further research on the effect of ethanol on other microfluidic channels with different configurations will shed more light on this finding. 

It is evident that the geometry of roughness elements could significantly alter the performance of the microfluidic devices. As a result, we suggest testing different geometries as a future research direction.

## Figures and Tables

**Table 1:** Geometrical parameters of all the devices tested (all the values are in µm except β, which is in degrees).

Device	L_1_ = L_2_ = L_3_	W_1_ = W_3_	W_2_	L_R_	H_R_	β
**Device 0**	2000	900	152	0	0	60°
**Device A**	2000	900	152	1/3 L_2_	0.1 W_2_	60°
**Device B**	2000	900	152	1/2 L_2_	0.1 W_2_	60°
**Device C**	2000	900	152	L_2_	0.1 W_2_	60°
**Device D**	2000	900	152	1/3 L_2_	0.01 W_2_	60°
**Device E**	2000	900	152	1/2 L_2_	0.01 W_2_	60°
**Device F**	2000	900	152	L_2_	0.0 1W_2_	60°

**Table 2:** The three design and optimization phases in this study.

-	Phase I	Phase II						Phase III	
Optimization strategy	Initial design	Utilization of wall roughness elements with different height (H_R_) and total length (L_R_)						Working fluid replacement with ethanol for the worst devices in Phase II	
Device	0	A	B	C	D	E	F	C	E
Inlet pressure (MPa)	---	2.06	3.10	3.10	2.06	2.48	1.86	1.76	2.06
